# Nafamostat mesilate attenuates renal fibrosis by suppressing the IL-17 signaling pathway

**DOI:** 10.3389/fphar.2025.1648623

**Published:** 2025-10-31

**Authors:** Weili Liao, Rui Fan, Yuman Du, Hairong Wang, Yong Yang, Yuan Tian

**Affiliations:** 1 Department of Nephrology, Jingzhou Hospital Affiliated to Yangtze University, Jingzhou, China; 2 School of Public Health, Mudanjiang Medical University, Mudanjiang, China

**Keywords:** renal interstitial fibrosis, Granzyme B, Nafamostat mesylate, tubular injury, inflammation

## Abstract

**Introduction:**

Chronic kidney disease (CKD) is a global public health concern characterized by progressive renal function decline and fibrosis, ultimately leading to end-stage renal disease (ESRD). Renal tubular injury and renal interstitial fibrosis are key contributor to this process. Granzyme B (GZMB), a serine protease, has been studied for its role in inducing apoptosis during immune defense. However, the role of GZMB in tubular injury and renal interstitial fibrosis remain unclear. Nafamostat mesylate (NM), a broad-spectrum serine protease inhibitor which is used for anticoagulation during hemodialysis in the clinic. This study aims to investigate the effects of GZMB on renal injury and renal interstitial fibrosis, and further explore the mechanisms of action NM intervention on renal injury and renal interstitial fibrosis.

**Method:**

To elucidate the therapeutic mechanisms of NM in renal fibrosis, we integrated *in vivo* unilateral ischemia-reperfusion injury (UIRI) models with *in vitro* experiments using human proximal tubular epithelial (HK-2) cells stimulated by TGF-β or GZMB. The therapeutic effect of NM was evaluated through renal function examination, histopathological assessment, immunofluorescence staining, Western blot and qRT-PCR analysis. In addition, RNA sequencning is conducted to identify key pathways. These methods collectively reveal the mechanisms both *in vivo* and *in vitro* by NM improves renal injury and fibrosis.

**Result:**

GZMB was upregulated in various mouse models of renal fibrosis as well as in TGF-β-stimulated HK-2 cells. *In vitro*, GZMB treatment induced HK-2 cell injury, inflammatory responses, and partial epithelial-mesenchymal transition (p-EMT). Transcriptomic analysis demonstrated that the combined administration of GZMB and perforin significantly altered the expression of genes associated with apoptosis, inflammation, and fibrosis. The serine protease inhibitor NM attenuated GZMB-induced HK-2 cell injury, inflammatory responses, and p-EMT. Furthermore, NM suppressed TGF-β-induced p-EMT. In a murine model of UIRI, NM administration improved renal function, reduced fibrotic deposition, and exerted protective effects against apoptosis and mitochondrial dysfunction. RNA-seq analysis suggested that the renoprotective effects of NM were mediated through inhibition of the IL-17/c-Fos signaling pathway.

**Discussion:**

This study confirmed that GZMB promotes the process of renal fibrosis by inducing renal tubular cell injury and p-EMT. NM can effectively antagonize the above-mentioned harmful effects induced by GZMB and TGF-β, and improve renal function and alleviate fibrosis in mouse models. Its renal protective effect is related to the inhibition of the IL-17/c-Fos signaling pathway. The above content proves that NM can be a potential drug for the treatment of CKD.

## Introduction

Chronic kidney disease (CKD) represents a significant global public health challenge, carrying a substantial disease burden with a worldwide prevalence reaching as high as 10% (Coresh et al., 2017). The core pathological hallmark of CKD is progressive renal function deterioration, which in its terminal stage advances to end-stage renal disease (ESRD) (Zhang et al., 2024). This process can be driven by mechanisms of tubular injury and renal interstitial fibrosis (Ram et al., 2022a). Recent studies have highlighted that renal tubular injury is a key driver of CKD progression. Damaged renal tubular epithelial cells can promote renal inflammation by secreting various pro-inflammatory cytokines (Liu et al., 2018), chemokines (Chang and Chen, 2020), reactive oxygen species (ROS) (Li et al., 2023), and C-reactive protein (CRP) (Pegues et al., 2016), among others. Additionally, injured renal tubular epithelial cells can produce multiple pro-fibrotic factors and trigger apoptosis, further exacerbating renal dysfunction (Liu et al., 2018; Jiang et al., 2022). Meanwhile, renal interstitial fibrosis is a central hallmark of CKD progression. It is characterized by excessive extracellular matrix (ECM) deposition secreted by activated myofibroblasts, which disrupts tubular structures and surrounding microvasculature, leading to irreversible scar formation (Humphreys, 2018; Yuan et al., 2022a). Renal tubular injury promotes fibrosis, while renal interstitial fibrosis, in turn, aggravates renal damage, forming a vicious cycle that ultimately accelerates the transition from CKD to ESRD.

Granzymes are key members of the serine protease family, including granzyme A, granzyme B, granzyme H, granzyme M, and granzyme K (Cigalotto and Martinvalet, 2024). They are primarily produced by cytotoxic T lymphocytes (CTLs) and natural killer (NK) cells, playing a critical role in immune defense mechanisms through granzyme-mediated cell death (Zhou et al., 2020). Among them, granzyme B (GZMB) is widely recognized for its ability to induce target cell death, primarily through classical apoptotic pathways. Upon entering the target cell cytoplasm, GZMB mediates mitochondria-dependent apoptosis by cleaving key pro-apoptotic proteins such as Bid and caspases. This process largely depends on the perforin-dependent pore delivery mechanism for cellular entry (Anthony et al., 2010; Prager et al., 2019). Li et al. (2024) found that human GZMB primarily exerts its antitumor effects by inducing apoptosis to lyse tumor cells. In addition to these cytotoxic effects, emerging evidence highlights the extracellular functions of GZMB. In inflammatory diseases, GZMB can promote inflammation by activating pro-inflammatory factors and degrading relevant ECM components (Velotti et al., 2020; Turner et al., 2021; Obasanmi et al., 2024). The continuous activation of multiple inflammatory mechanisms, such as immune cell response, NLRP3 inflammasome activation and oxidative stress response, can jointly promote apoptosis, necrosis and interstitial fibrosis of renal tubular epithelial cells (Jha et al., 2021). Previous research on GZMB has mainly focused on its role in inflammation and tumors, while its function in renal tubular injury and renal interstitial fibrosis remains unclear.

Nafamostat mesylate (NM) is a broad-spectrum serine protease inhibitor that primarily exerts its anticoagulant effect through thrombin inhibition in renal diseases, and it is predominantly used for anticoagulation during continuous renal replacement therapy (CRRT) in the clinic (Duan et al., 2018; Chen et al., 2023). Growing evidence suggests that serine protease inhibitors can promote the development and progression of kidney diseases by influencing inflammation, fibrosis, and other processes (Morinaga et al., 2013; Bronze-da-Rocha and Santos-Silva, 2018). However, research on NM has primarily focused on its therapeutic effects on systemic inflammatory diseases (Yates et al., 2022), while exploration of its impact on kidney diseases remains relatively limited.

In this study, both *in vivo* renal fibrosis models and HK-2 cells treated with TGF-β confirmed that the expression of GZMB was significantly upregulated during disease progression. *In vitro* studies revealed that GZMB induced cells damage, inflammatory response, p-EMT in HK-2 cells. Furthermore, GZMB induced tubular epithelial cell death in perforin-dependent and independent way. Notably, NM demonstrated significant renal protective effects and anti-fibrotic activity, suggesting its potential value as a novel therapeutic strategy for CKD. Mechanistically, NM inhibited the activation of IL-17/c-Fos signaling. Our study provides a therapeutic target, GZMB and a novel drug, NM for the treatment of CKD.

## Methods

### Animal models

All C57BL/6J mice used in this study were purchased from Beijing Vital River Laboratory Animal Technology Co., Ltd. The mice were male, 8 weeks, and housed under standard laboratory conditions with a 12-h light/dark cycle, temperature maintained at 22 °C–25 °C, and humidity at 40%–60%. They had free access to food and water. The mice were randomly divided into three groups: (I) Control group; (II) UIRI group; (III) UIRI + NM treatment group. The UIRI (unilateral ischemia-reperfusion injury) kidney model was established as follows: under general anesthesia, a midline abdominal incision was made, and the left renal pedicle was clamped for 35 min using a vascular clip. During ischemia, mice were kept in a thermostatic chamber at 38 °C. After 35 min, the clip was removed, and the abdomen was sutured layer by layer. NM (HY-B0190A, MCE) was dissolved in saline at a concentration of 10 mg/kg (Yan et al., 2022). Intraperitoneal administration began on day 4 post-UIRI and continued for 7 days. On day 10, the contralateral kidney was removed, and on day 11, the mice were euthanized for blood and kidney sample collection. The unilateral ureteral obstruction (UUO) mouse model was established with male C57BL/6J; 129 mice at 8 weeks of age. The left ureter of mice was exposed through the left incision and ligated with 4–0 silk thread. The sham operation group only received kidney exposure surgery and did not undergo ligation surgery. The left kidney was collected 7 days after the operation. The folic acid-induced nephropathy (FAN) mouse model was established with male C57BL/6J; 129 mice at 8 weeks of age. Renal injury was induced by intraperitoneal injection of 200 mg/kg folic acid dissolved in 0.3M NaHCO3. Mice in the control group only received intraperitoneal injection of 0.3M NaHCO3. The kidneys of the mice were collected 28 days after injection.

### Cells culture and treatment

Human proximal tubular epithelial cells (HK-2) were cultured in DMEM/F12 medium supplemented with 10% fetal bovine serum (FBS) and 1% antibiotics (penicillin-streptomycin) at 37 °C under 5% CO_2_. Three experimental treatments were performed: (I)TGF-β Stimulation Assay: HK-2 cells were inoculated in six-well plates and stimulated for 24 h in serum-free medium with TGF-β (2.5 ng/mL) (P01137, R&D Systems), and were divided into the control group and the TGF-β group. (II)NM Intervention Assay: The HK-2 cells cultured *in vitro* were co-incubated with TGF-β and NM (100 μM) in serum-free medium for 24 h and were divided into: the control group, the TGF-β group and the TGF-β + NM group. (III)GZMB Stimulation Assay: In serum-free medium, HK-2 cells were stimulated with GZMB cytokines (5 μg/mL) (Shen et al., 2016) (ab307481, Abcam) for 24 h and were divided into: the control group and the GZMB group. (IV) GZMB and NM Intervention Assay: The HK-2 cells cultured *in vitro* were co-incubated with GZMB and NM in serum-free medium for 24 h and were divided into: the control group, the GZMB group and the GZMB + NM group.

### Western blot analysis

Protein lysates from HK-2 cells or renal tissues were extracted using RIPA buffer supplemented with PMSF, protease inhibitors, and phosphatase inhibitors. Protein concentrations were quantified using a BCA protein assay kit. Protein samples were separated by electrophoresis on 8%, 10%, or 12% SDS-PAGE gels and subsequently transferred to PVDF membranes. The membranes were blocked with 5% non-fat milk and incubated overnight at 4 °C with primary antibodies (see Supplementary Table S1). After washing, the membranes were incubated with horseradish peroxidase (HRP)-conjugated anti-mouse or anti-rabbit IgG secondary antibodies for 1 h at room temperature. Protein signals were detected using enhanced chemiluminescence (ECL) substrate. Western blot images were compiled using Adobe Photoshop, quantified with ImageJ software (v1.8.0), and statistically analyzed using GraphPad Prism 8.

### RNA extraction and qRT-PCR analysis

Total RNA was extracted from HK-2 cells and mouse renal tissues using TRIzol reagent (Takara). cDNA was synthesized using the HiScript II Reverse Transcription System (Vazyme, Nanjing, China). Quantitative real-time PCR (qRT-PCR) was performed using the ChamQ SYBR Color qPCR Master Mix kit (Vazyme). Primer sequences are provided in Supplementary Table S2.

### Cell viability assay

The impact of GZMB and perforin co-treatment on cell viability was assessed using the CCK-8 assay. HK-2 cells were seeded in 96-well plates for 24 h, followed by 24-h co-incubation with GZMB (2 μg/mL) and perforin (160 ng/mL). According to the manufacturer’s protocol (CCK-8 kit, C0037, Beyotime), 100 μL of culture medium containing 10% CCK-8 reagent was added to each well. The plates were then incubated at 37 °C for 2 h. Absorbance was measured at 450 nm using a microplate reader.

### Renal function analysis

Collecting 1.5 mL of whole blood from mouse and keeping it at 4 °C overnight. On the following day, separating the serum by centrifuging the sample at 4 °C, 3000rpm for 10 min to isolate the serum. Withdrawing 30 μL for Serum creatinine (Scr) measurement and storing the remaining serum at −80 °C for future use. Scr levels were measured using the DICT-500 system (Bioassay Systems, Hayward, CA, United States). Blood Urea Nitrogen (BUN) levels were measured using the corresponding kits (NO.C013-2-1) (Nanjing Jiancheng Company, China).

### Immunofluorescence staining

HK-2 cells were plated on coverslips and cultured for 24 h. The medium was then replaced with serum-free basal medium, and cells were treated with TGF-β (2.5 ng/mL) and NM (100 μM), categorized into control, TGF-β, and TGF-β + NM groups. Subsequent experiments were conducted after 24h of treatment. HK-2 cells were grown on coverslips and fixed with pre-chilled methanol for 15 min. The cell-covered coverslips were then incubated overnight at 4 °C with primary antibodies against fibronectin and α-SMA. The following day, samples were incubated for 1 h at room temperature with donkey anti-rabbit (H + L) secondary antibody (light-protected conditions), followed by nuclear staining with DAPI. Fluorescence images were acquired using a LSM780 confocal laser scanning microscope system (ZEISS, Oberkochen, Germany).

### Histopathological evaluation

Renal tissue samples were fixed in 4% paraformaldehyde and embedded in paraffin. Sections (3 μm thick) were deparaffinized, rehydrated, and subjected to Masson’s trichrome staining to assess collagen deposition and fibrotic lesions. Quantitative analysis of fibrotic lesions was performed using ImageJ software (v1.8.0), with at least three randomly selected images analyzed per mouse.

### Hematoxylin–eosin (H&E) staining

The renal tissue sections were dewaxed and rehydrated into water through a series of graded alcohols. The sections were stained with hematoxylin for 10 min, then rinsed under running tap water to remove excessive staining agent. After differentiation and bluing, rinsed again. Then, re-stain with eosin staining solution for 50s. Seal the sheet after dehydration and cleaning. Finally, morphological observations were conducted using an optical microscope (Motic China Group Co., Ltd., China).

### RNA sequencing (RNA-seq) and analysis

Total RNA was extracted from HK-2 cells and kidney tissues of mice subjected to ischemia-reperfusion and NM treatment using TRIzol^®^ Reagent (Thermo Fisher, Cat#15596026). The purity and concentration of RNA were measured using a NanoDrop 2000 spectrophotometer, while RNA integrity was assessed with an Agilent 2100 Bioanalyzer/LabChip. After confirming sample quality, library construction was performed as follows: eukaryotic mRNA was enriched using magnetic beads with Oligo (dT) primers, followed by fragmentation into 200–300 bp segments. First-strand cDNA and second-strand cDNA were synthesized using mRNA as a template, purified, and subjected to end repair, A-tailing, and adapter ligation. Finally, PCR amplification was performed to enrich the cDNA library. Quality control was conducted to ensure library integrity. Sequencing was then carried out on the Illumina NovaSeq 6,000 platform using a PE150 (Paired-End 150 bp) sequencing mode. Finally, data analysis was conducted, DEGs were identified using DESeq2 or edgeR, with a significance threshold of p < 0.05. Functional annotation of DEGs was performed through GO analysis, while pathway enrichment was assessed via KEGG analysis using Cluster Profiler.

### Statistical analysis

All data are presented as Mean ± SD. Data analysis and visualization were performed using GraphPad Prism (GraphPad Software, San Diego, CA, United States). Two-tailed Student’s unpaired t-test analysis was utilized to compare between two groups. One-way ANOVA followed by LSD or Dunnett post-test was performed to compare more than three groups. A p-value <0.05 was considered statistically significant. All experiments were performed with at least three independent replicates.

## Result

### GZMB was upregulated in various mouse models of renal fibrosis and HK-2 cells treated with TGF-β

**FIGURE 1:**
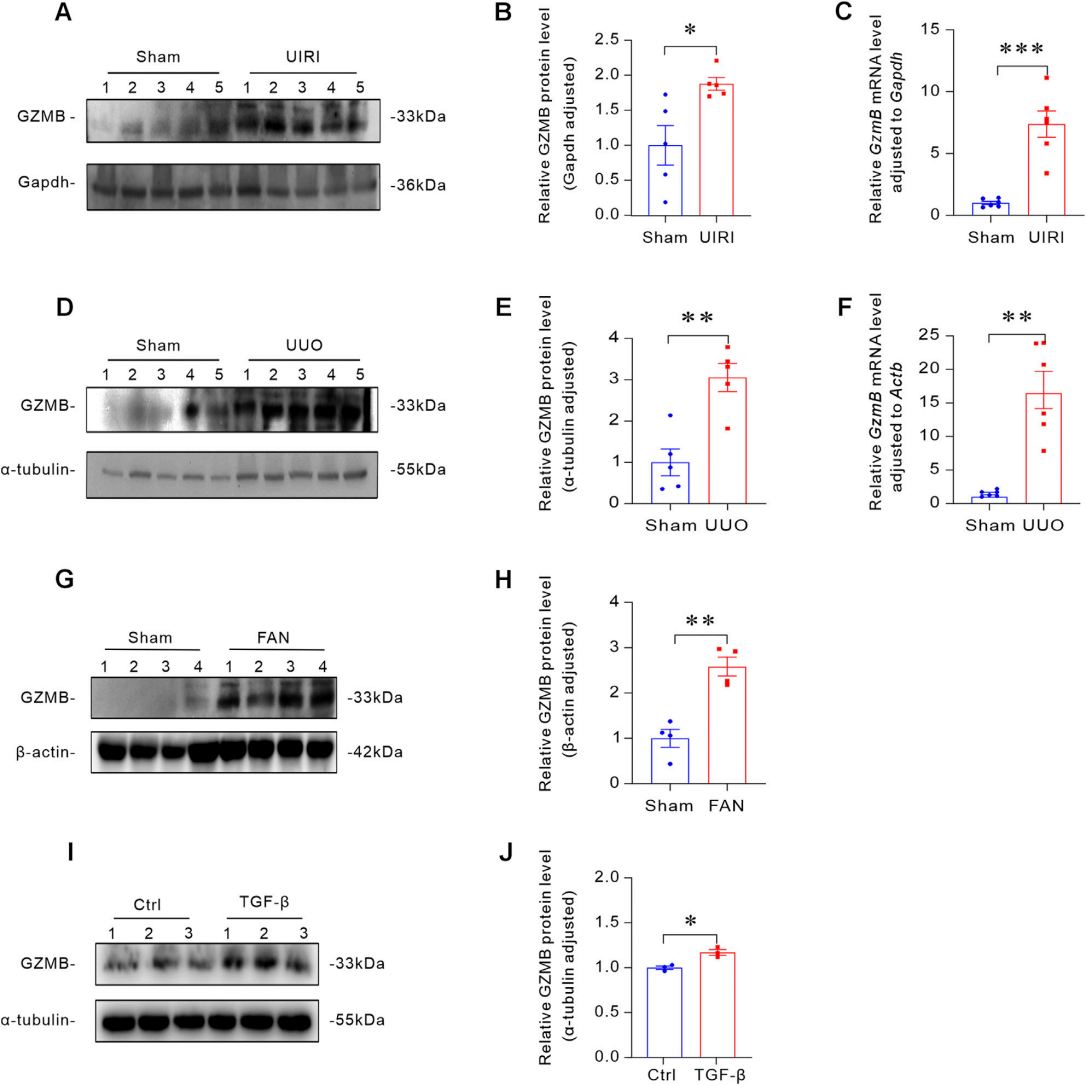
GZMB was upregulated in various mouse models of renal fibrosis and HK-2 cells treated with TGF-β. **(A,B)** Representative Western blot **(A)** and quantitative analysis **(B)** of GZMB protein levels in the kidneys of different groups (n = 5 biologically independent mice). **(C)** Relative mRNA levels of *GzmB* in the kidneys of sham-operated and UIRI group mice (n = 6 biologically independent mice). **(D,E)** Representative Western blot **(D)** showing renal expression of GZMB and quantitative analysis **(E)** of GZMB (n = 5 biologically independent mice). **(F)** Relative mRNA levels of *GzmB* (n = 6 biologically independent mice). **(G,H)** Representative Western blot **(G)** and quantitative data **(H)** of GZMB in the kidneys of sham-operated and FAN group mice (n = 4 biologically independent mice). **(I,J)** TGF-β induces GZMB protein expression in HK-2 cells. Representative Western blot **(I)** and quantitative data **(J)** after HK-2 cells were incubated with TGF-β (2.5 ng/mL) for 24 h (n = 3 per group). *p < 0.05, **p < 0.01, ***p < 0.001. Data are presented as Mean ± SD, conforming to normal distribution and homogeneity of variance. Two-tailed Student’s unpaired t-test analysis was utilized to compare between two groups.

To investigate the role of GZMB in renal fibrosis, we established multiple murine models of kidney fibrosis. In the UIRI-induced renal fibrosis model, Western blot and qRT-PCR analyses revealed significant upregulation of *GzmB* in fibrotic kidneys compared to controls (Figures 1A–C). Meanwhile, UUO operation was performed on mice, and it was found that the expression levels of *GzmB* protein and mRNA in the kidneys of this model increased (Figures 1D–F). We also further analyzed through Western blot and found that the FAN model enhanced the expression of GZMB in the kidneys of mice (Figures 1G,H). To further explore the effect of fibrosis on the expression of GZMB *in vitro*, we stimulated human proximal renal tubular (HK-2) cells with TGF-β, a key mediator of renal fibrosis, to induce the occurrence of fibrosis. The Western blot results indicated that TGF-β stimulation increased the protein level of GZMB (Figures 1I,J). These findings suggest that GZMB is a potential participant in the pathogenesis of renal fibrosis.

### GZMB induced cell damage, inflammatory responses, p-EMT of tubular epithelial cells

**FIGURE 2:**
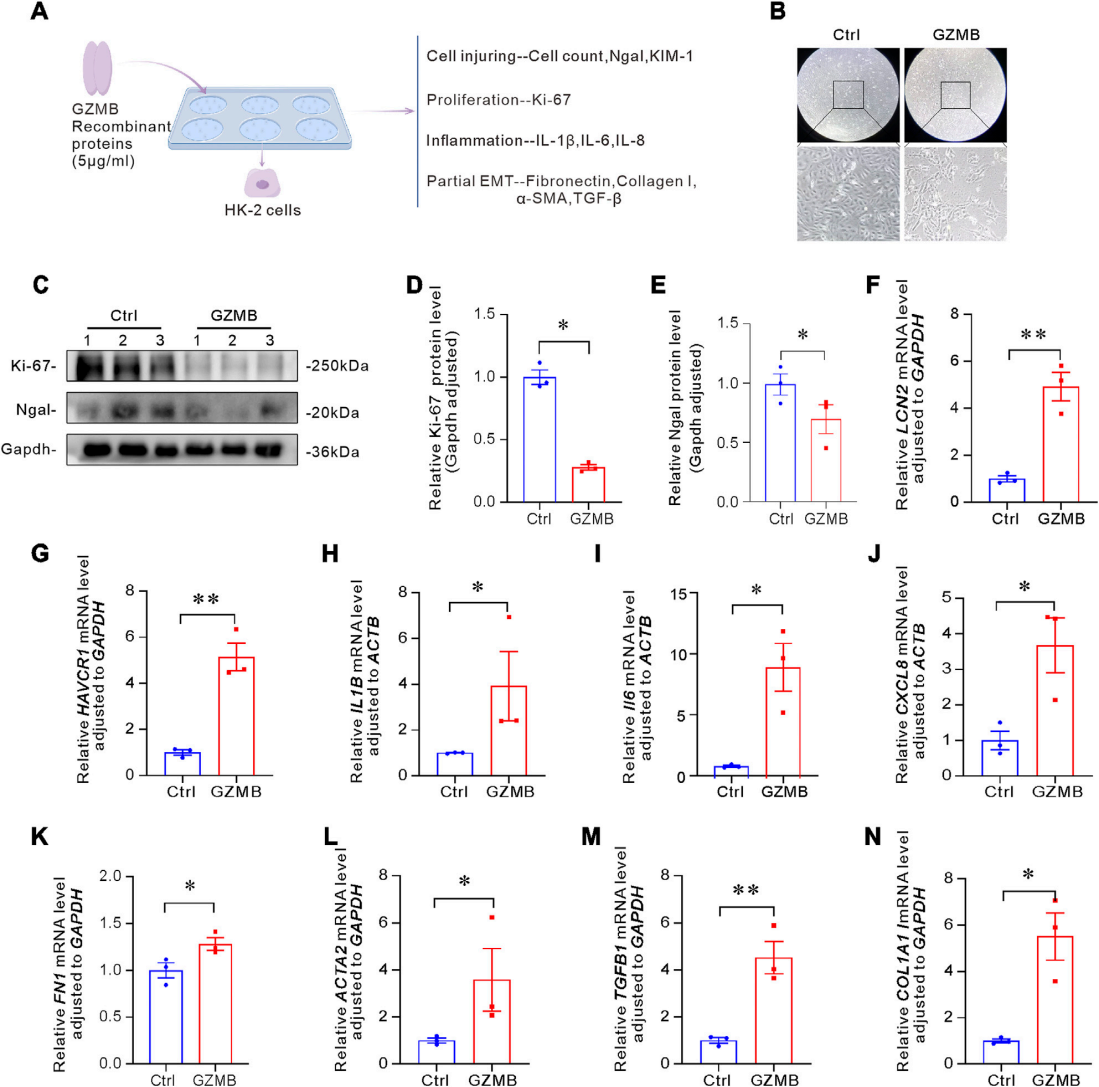
GZMB induced cells damage, inflammatory response, p-EMT of tubular epithelial cells. **(A)** Schematic diagram of the experimental design. HK-2 cells were incubated with human recombinant GZMB protein (5 μg/mL) for 24 h, followed by evaluation of cell injury, proliferation, inflammation, and partial EMT-related markers. **(B)** Bright-field images of HK-2 cells and cells level in the control group and after 24-h stimulation with GZMB. **(C–E)** Effects of GZMB on proliferation and renal injury markers. Representative Western blot **(C)** and quantitative analysis of Ki-67 **(D)** and Ngal **(E)** (n = 3 per group). **(F,G)** qRT-PCR analysis of *LCN2* **(F)** and *HAVCR1* **(G)** mRNA expression (n = 3 per group). **(H–J)** Relative mRNA levels of pro-inflammatory cytokines *IL1B* **(H)**, *IL6* **(I)** and *CXCL8* **(J)** (n = 3 per group). (K–N) Relative mRNA levels of *FN1* **(K)**, *ACTA2* **(L)**, *TGFB1* **(M)** and *COL1A1* **(N)** in HK-2 cells after 24-h GZMB stimulation (n = 3 per group). *p < 0.05, **p < 0.01. Data are presented as Mean ± SD. Statistical significance was determined by Two-tailed Student’s unpaired t-test analysis between two groups.

To explore the effects of the human recombinant protein GZMB on cells, we added GZMB to HK-2 cells for cultivation. After 24 h, we evaluated the damage, proliferation, inflammation, and p-EMT of HK-2 cells (Figure 2A). Dual-field microscopy revealed that compared to the control group, the number of HK-2 cells treated with GZMB was significantly reduced (Figure 2B). The decline ratio can reach 56%. To further confirm the cytotoxic effects of GZMB, we analyzed the expression of Ki-67, a cell proliferation marker, and Ngal, a kidney injury marker, via Western blot, as shown in Figures 2C–E, the protein expression levels of Ki-67 and Ngal in GZMB-treated cells significantly decreased, indicating that GZMB treatment inhibited cell proliferation and exacerbated cell damage. Meanwhile, we also detected the mRNA expression of *LCN2* and *HAVCR1*, both of which consistent results were obtained (Figures 2F,G). Next, we examined the effect of GZMB on the expression of pro-inflammatory factors in HK-2 cells. qRT-PCR results demonstrated that the mRNA levels of *IL1B*, *IL6* and *CXCL8* were significantly upregulated, suggesting that GZMB triggers an inflammatory response in human renal tubular epithelial cells (Figures 2H–J). Additionally, to assess the potential role of GZMB in p-EMT, we analyzed related genes. GZMB treatment significantly increased the mRNA levels of *FN1*, *ACTA2*, *TGFB1* and *COL1A1*, indicating that GZMB promotes fibrotic responses in HK-2 cells (Figures 2K–N). In summary, these findings demonstrate that GZMB exacerbates cell damage, induces the release of inflammatory factors, and contributes to the progression of p-EMT.

### GZMB induced tubular epithelial cells damage in perforin-dependent way

**FIGURE 3:**
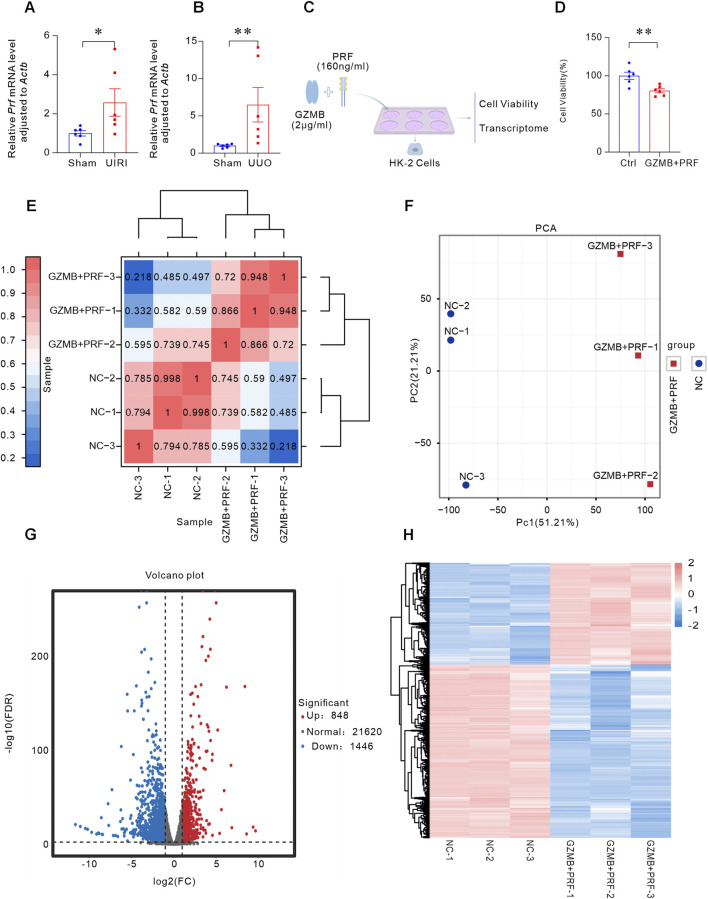
GZMB induced tubular epithelial cells damage in perforin-dependent way. **(A)** *Prf* mRNA expression levels in the kidneys of UIRI mouse models (n = 6 biologically independent mice). **(B)** Detection of *Prf* in UUO mouse kidneys by qRT-PCR (n = 6 biologically independent mice). **(C)** Experimental design: HK-2 cells were co-incubated with GZMB (2 μg/mL) and PRF (160 ng/mL), followed by cell viability assessment and transcriptome sequencing analysis. **(D)** Cell viability monitored by CCK-8 assay. **(E)** Pearson correlation heatmap between samples. The color scale represents correlation coefficients between samples (closer to 1 indicates stronger correlation). **(F)** PCA showing transcriptional differences between the GZMB + PRF group (red dots) and NC group (blue dots) (PC1 = 51.21%, PC2 = 21.21%). **(G)** Volcano plot of DEGs between control and treatment groups. The X-axis represents log2(FC), and the Y-axis represents -log10(FDR). Blue dots: downregulated genes; red dots: upregulated genes; gray dots: non-significant genes. **(H)** Hierarchical clustering heatmap of DEGs. Each column represents a sample, and each row represents a gene. Colors are scaled based on log10(FPKM+0.000001), with darker shades indicating higher expression. *p < 0.05, **p < 0.01. Data are presented as Mean ± SD. Statistical analysis was performed using two-tailed Student’s unpaired t-test analysis.

Since GZMB exerts its pro-apoptotic function in a perforin-dependent way, we examined the expression of perforin in fibrotic kidneys. In two classical kidney fibrosis models, we observed a significant increase in the expression of *Prf* gene (Figures 3A,B). To verify whether GZMB interacts with perforin, we co-cultured HK-2 cells with both GZMB and perforin in the medium and assessed cell viability using the CCK-8 assay. The results showed that the combined treatment led to a significant decrease in cell viability (Figures 3C,D), indicating that GZMB-mediated cytotoxicity in a perforin-dependent way.

To further reveal the gene expression changes induced by the combined action of GZMB and perforin through transcriptomic analysis, we performed RNA-seq on HK-2 cells co-incubated with GZMB and perforin compared to control cells. The Pearson correlation heatmap showed that the correlation coefficients between intra-group samples were close to 1, confirming good experimental reproducibility. While the transcriptomic profile in negative control (NC) group significantly differed from the GZMB and PRF treated (GZMB + PRF) group (Figure 3E). Principal component analysis (PCA) further supported these findings, revealing low intra-group variation and high reproducibility in both GZMB + PRF and NC groups, while a significant difference was observed between NC and GZMB + PRF groups along PC1 (51.21%) (Figure 3F). Volcano plot analysis identified 2,294 differentially expressed genes (DEGs) meeting the threshold criteria (FDR<0.05, |log2(FC)|>1), including 848 upregulated and 1,446 downregulated genes (Figure 3G). Additionally, a heatmap of DEGs expression further demonstrated that samples from the same treatment group exhibited similar gene expression patterns, while significant differences were observed between NC and GZMB + PRF groups (Figure 3H).

**FIGURE 4:**
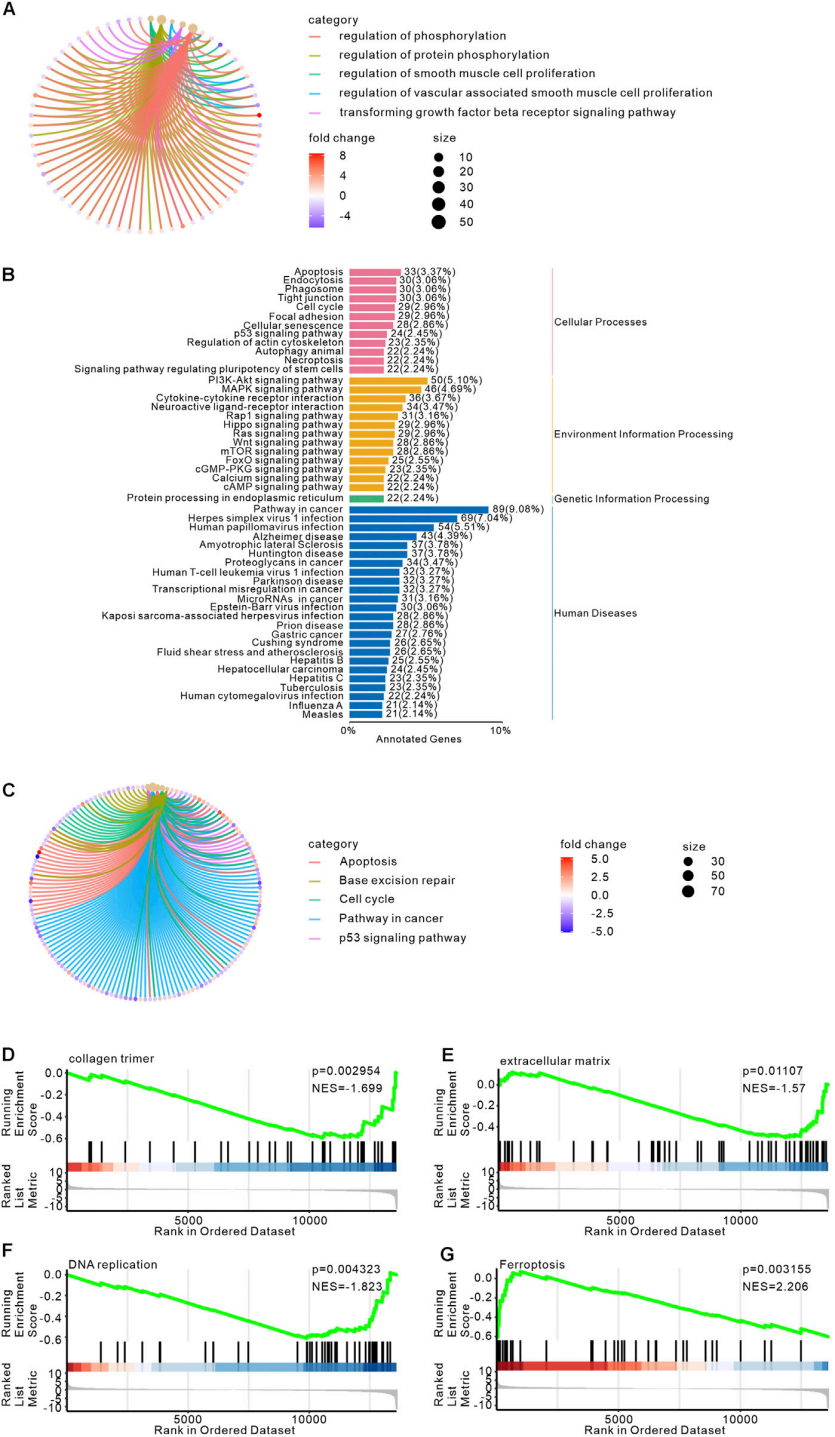
Bioinformatics analysis of transcriptome sequencing in HK-2 cells treated with GZMB and perforin. **(A)** GO enrichment network for biological regulation. The network illustrates the top 5 significantly enriched pathways and associated genes in biological regulation. Line colors denote different pathways, gene node colors reflect fold changes, and pathway node sizes correspond to the number of enriched genes (larger nodes indicate more genes). **(B)** KEGG classification of DEGs. The x-axis shows the number of genes annotated to each pathway and their proportion relative to all annotated genes. The left y-axis lists KEGG pathway names, while the right y-axis categorizes metabolic pathways: pink (Cellular Processes), yellow (Environmental Information Processing), green (Genetic Information Processing), and blue (Human Diseases). **(C)** Network of DEGs and KEGG pathways. The network highlights the top 5 enriched KEGG pathways, with line colors representing pathways, gene node colors indicating fold changes, and pathway node sizes reflecting the number of enriched genes. **(D–G)** GSEA results plot. Plot titles specify GO Term/KEGG Pathway names: collagen trimer **(D)**, extracellular matrix **(E)**, DNA replication **(F)**, and ferroptosis **(G)**. The x-axis represents ranked gene positions (black vertical lines mark individual genes). The upper y-axis shows running enrichment scores (green curves), where positive ES values indicate upregulated trends and negative ES values denote downregulation.

We performed functional annotation and enrichment analysis on the identified differentially expressed genes (DEGs). For the differentially expressed genes (DEGs) enriched in biological processes, we further constructed a network diagram, which revealed that the DEGs were primarily clustered in regulation of smooth muscle cell proliferation, transforming growth factor beta receptor signaling pathway, indicating p-EMT (Figure 4A). Furthermore, KEGG pathway analysis categorized the enriched DEG pathways into four major branches: Cellular Processes, Environmental Information Processing, Genetic Information Processing, and Human Diseases. Within Cellular Processes, apoptosis-related DEGs constituted the largest subset (33 genes), followed by endocytosis, phagosome, and tight junction pathways. In Environmental Information Processing, the PI3K-Akt signaling pathway contained the highest number of associated genes (50 genes), with the MAPK signaling pathway ranking second. For Genetic Information Processing, protein processing in the endoplasmic reticulum was the dominant pathway (22 genes). In Human Diseases, cancer-related DEGs were most abundant (89 genes), followed by Herpes simplex virus 1 infection. These findings collectively indicate that most GZMB-activated pathways are implicated in regulating cellular damage, apoptosis, and inflammatory responses (Figure 4B). The KEGG enrichment network analysis of differentially expressed genes (DEGs) revealed their predominant enrichment in the Pathways in cancer (Figure 4C), these findings strengthen the association between GZMB/PRF and the regulation of apoptosis and cell cycle. We performed Gene Set Enrichment Analysis (GSEA)-based GO biological pathway analysis were primarily enriched in collagen trimer (Figure 4D) and extracellular (Figure 4E). GSEA-based KEGG pathway analysis revealed significant downregulation of DNA replication-related genes (Figure 4F), while demonstrating marked upregulation of ferroptosis-associated gene sets (Figure 4G). These results demonstrate that the differentially expressed genes are predominantly associated with apoptosis, inflammation, and fibrotic signaling, along with a distinct disruption of extracellular matrix homeostasis. These findings cell damage characterized by apoptosis, p-EMT, and ferroptosis in PRF abundant.

### NM alleviated cell damage, inflammatory responses, p-EMT of tubular epithelial cells induced by GZMB

**FIGURE 5:**
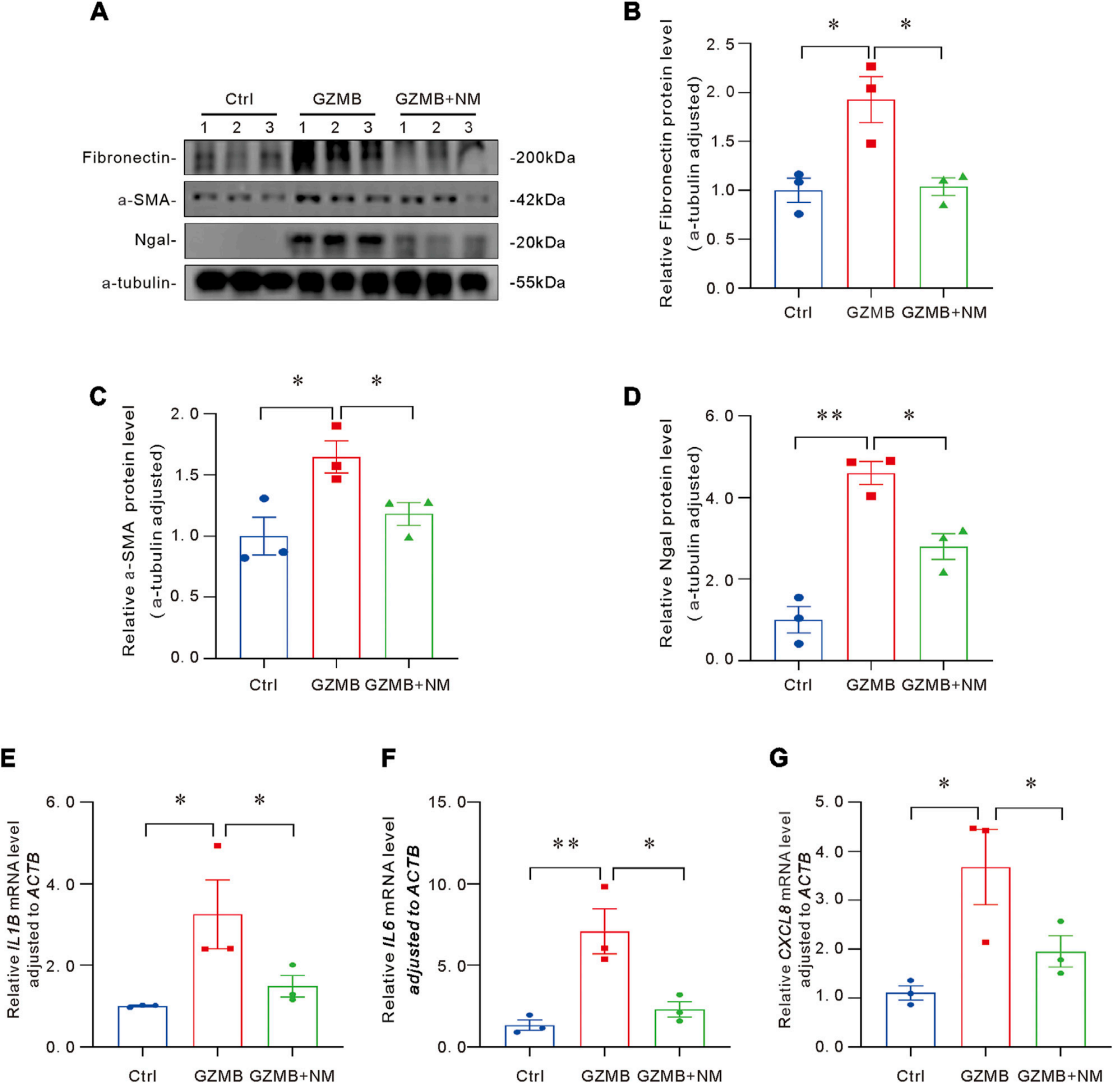
NM alleviated p-EMT, cell damage and inflammatory response induced by GZMB. **(A–D)** Representative Western blot **(A)** and quantitative analysis of Fibronectin **(B)**, α-SMA **(C)** and Ngal **(D)** protein levels in the kidneys of different groups (n = 3 per group). **(E–G)** Relative mRNA levels of pro-inflammatory cytokines *IL1B* **(E)**, *IL6* **(F)** and *CXCL8* **(G)** (n = 3 per group). *p < 0.05, **p < 0.01, ***p < 0.001. Data are presented as Mean ± SD. Intergroup differences were analyzed by one-way ANOVA followed by LSD *post hoc* tests.

To further investigate whether NM could ameliorate GZMB-induced p-EMT, cell damage, and inflammatory responses, HK-2 cells were co-cultured with NM and GZMB. Western blot analysis demonstrated that the expression levels of p-EMT-associated markers, including Fibronectin and α-SMA, as well as the renal injury biomarker Ngal, were significantly reduced in the NM-treated group (Figures 5A–D). These findings demonstrated that NM effectively mitigates the p-EMT process in tubular cells and alleviates cell damage. Additionally, to assess the potential anti-inflammatory effects of NM, we examined the expression of inflammation-related genes such as *IL1B*, *IL6*, and *CXCL8*. The results showed a notable decrease in the expression levels of these genes following NM treatment (Figures 5E–G), indicating that NM can effectively suppress the production of inflammatory cytokines. In summary, these results demonstrate that NM can alleviate GZMB-induced cellular injury, inhibit inflammatory cytokine release, and slow the progression of p-EMT.

### NM attenuated p-EMT of HK-2 cells induced by TGF-β

**FIGURE 6:**
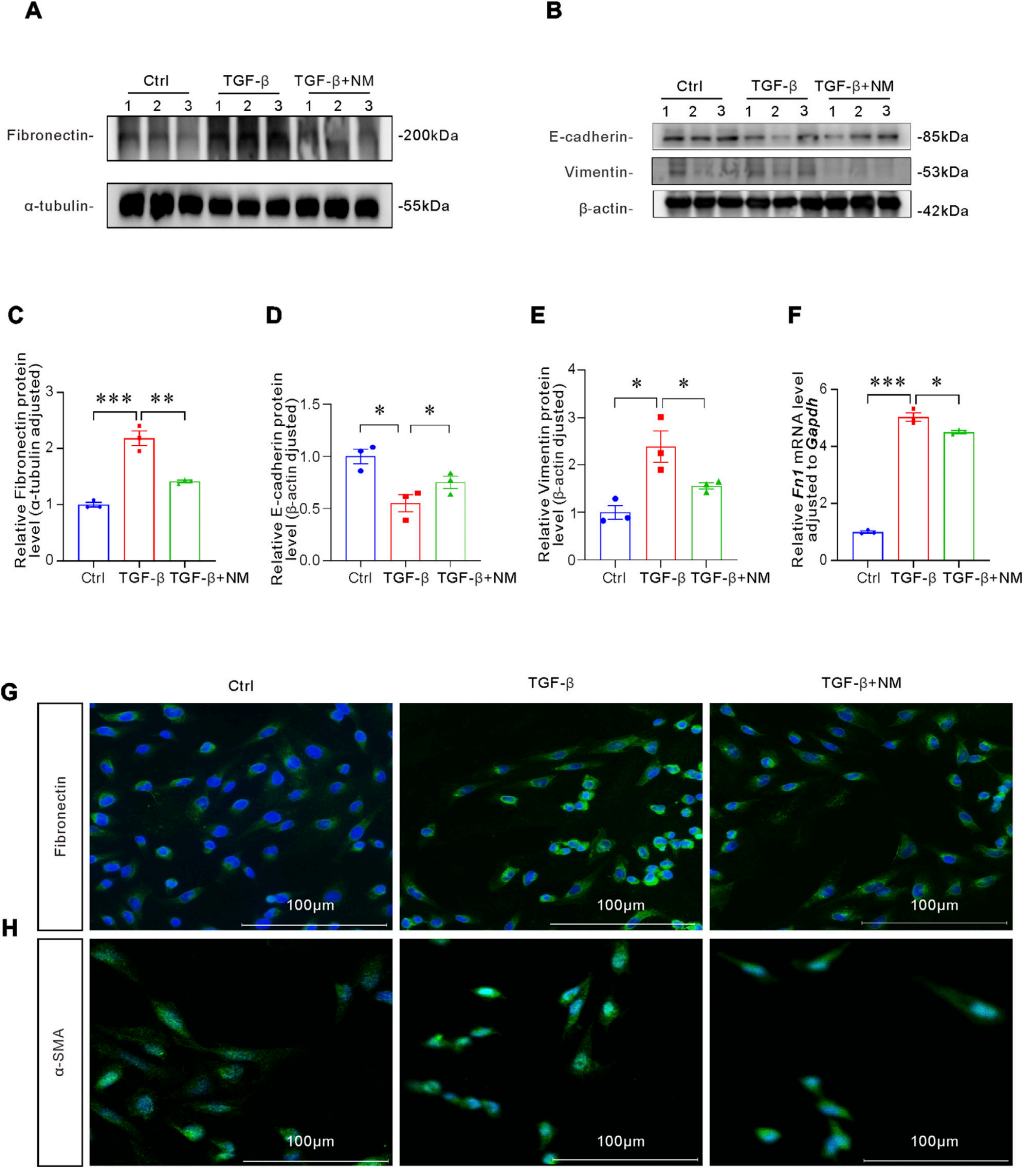
NM inhibited TGF-β-induced p-EMT process of HK-2 cells. **(A–E)** Representative Western blot of Fibronectin **(A)**, E-cadherin and Vimentin **(B)** and quantitative analysis of Fibronectin **(C)**, E-cadherin **(D)** and Vimentin **(E)** (n = 3 per group). **(F)** *FN1* expression levels evaluated by qRT-PCR (n = 3 per group). **(G,H)** IF staining of Fibronectin **(G)** and α-SMA **(H)** showing NM’s inhibition of TGF-β-induced p-EMT. Scale bar: 100 μm *p < 0.05, **p < 0.01, ***p < 0.001. Data are presented as Mean ± SD. Significance was determined by one-way ANOVA followed by LSD *post hoc* test.

We have previously demonstrated that GZMB, a serine protease, induces renal tubular injury. To further explore regulatory mechanisms, we analyzed the impact of the serine protease inhibitor NM on renal injury progression. To determine the potential protective effect of NM in renal p-EMT process, we co-cultured HK-2 cells with TGF-β in the presence or absence of NM. CCK8 found that compared with the control group, NM with a concentration of 100 μM had no significant effect on cell activity (Supplementary Figure S1A). Western blot demonstrated that NM treatment can improved the expression of Fibronectin, E-cadherin and Vimentin induced by TGF-β (Figures 6A–E). Meanwhile, qRT-PCR, and IF staining consistently demonstrated that NM treatment significantly suppressed TGF-β-induced fibronectin expression (Figures 6F,G). TGF-β, a key cytokine implicated in EMT (Ram et al., 2022b), significantly upregulated fibronectin expression in HK-2 cells. Importantly, administration of the serine protease inhibitor NM attenuated TGF-β-induced p-EMT. During EMT, some transformed mesenchymal cells differentiate in α-SMA-positive myofibroblasts, which excessively produce ECM (Yuan et al., 2022b). As a core marker of myofibroblasts, the expression level of α-SMA indirectly reflects the progression of EMT. Furthermore, NM treatment markedly downregulated α-SMA expression in TGF-β-stimulated HK-2 cells (Figure 6H), suggesting that NM may downregulate fibronectin expression by inhibiting myofibroblasts activation. In summary, NM exerts potential anti-fibrotic effects by inhibiting the TGF-β signaling pathway and suppressing the expression of fibrotic markers in renal tubular epithelial cells.

### NM alleviated renal fibrosis after ischemia-reperfusion injury in mice

**FIGURE 7:**
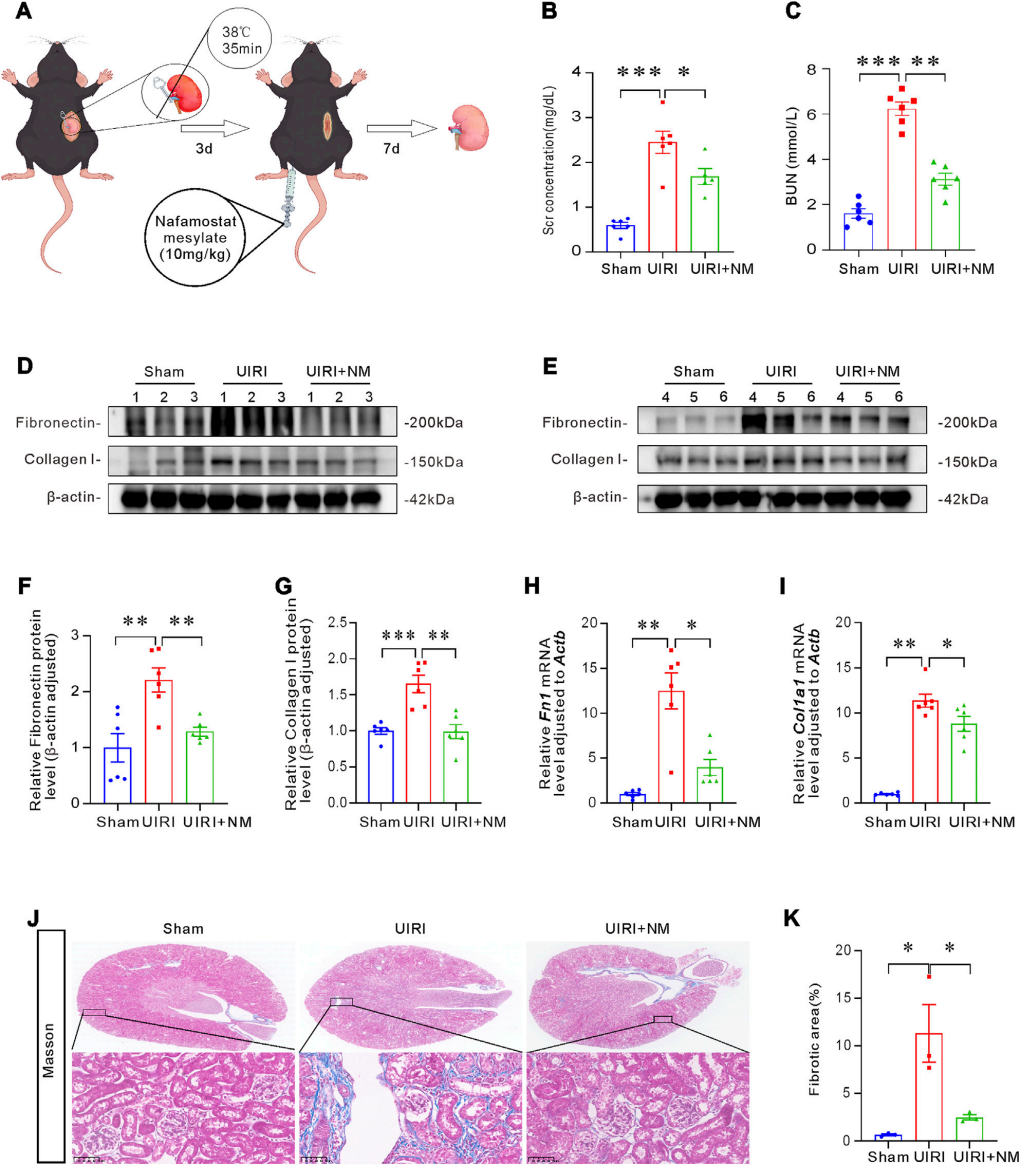
NM ameliorated renal fibrosis in UIRI mice. **(A)** Schematic diagram of the UIRI mouse model establishment and the NM administration protocol (10 mg/kg) in UIRI mice. **(B,C)** Quantitative analysis of Scr **(B)** and BUN **(C)** levels. **(D,E)** Representative Western blot images of Fibronectin and Collagen I, with numbers (1-6) indicating individual mice within each group. **(F,G)** Quantitative data of Fibronectin **(F)** and Collagen I **(G)** protein expression in different groups (n = 6 biologically independent mice). **(H,I)** mRNA levels of *Fn1* **(H)** and *Col1a1* **(I)** in kidney tissues across groups (n = 6 biologically independent mice). **(J)** Representative Masson’s trichrome-stained micrographs showing NM-mediated attenuation of renal fibrotic lesions in UIRI mice. Blue areas indicate collagen deposition (scale bar: 50 μm). **(K)** Quantitative analysis of renal fibrotic lesions (n = 3 biologically independent mice). *p < 0.05, **p < 0.01, ***p < 0.001. Data are presented as Mean ± SD. Intergroup differences were analyzed by one-way ANOVA followed by Dunnett *post hoc* tests.

Given NM’s demonstrated efficacy in mitigating renal fibrosis by blocking TGF-β signaling *in vitro*, we employed a murine UIRI model to evaluate its potential *in vivo* effects. In the UIRI model, daily intravenous NM administration was initiated at D4 post-surgery, followed by contralateral nephrectomy at D10 and sample collection at D11 (Figure 7A). Renal function assessment revealed that while UIRI induced elevated Scr and BUN levels, NM treatment significantly attenuated this increase (Figures 7B,C). Next, we evaluated the effect of NM treatment on renal fibrosis in mice. As shown in Figures 7D,E, NM ameliorated UIRI-induced upregulation of Fibronectin and Collagen I expression, with quantitative analysis of their protein levels presented in Figures 7F,G mRNA expression analysis of fibrotic markers yielded consistent results with the protein expression patterns (Figures 7H,I). Masson’s trichrome staining revealed substantial collagen deposition in renal interstitium following UIRI, which was significantly attenuated by NM treatment (Figures 7J,K). Collectively, these *in vivo* findings demonstrate that NM exerts potent renoprotective and anti-fibrotic effects by modulating ECM remodeling and suppressing key fibrotic factors, thereby mitigating the progression of ischemic injury-induced renal fibrosis.

### NM demonstrated renal protective effects in UIRI mice

**FIGURE 8:**
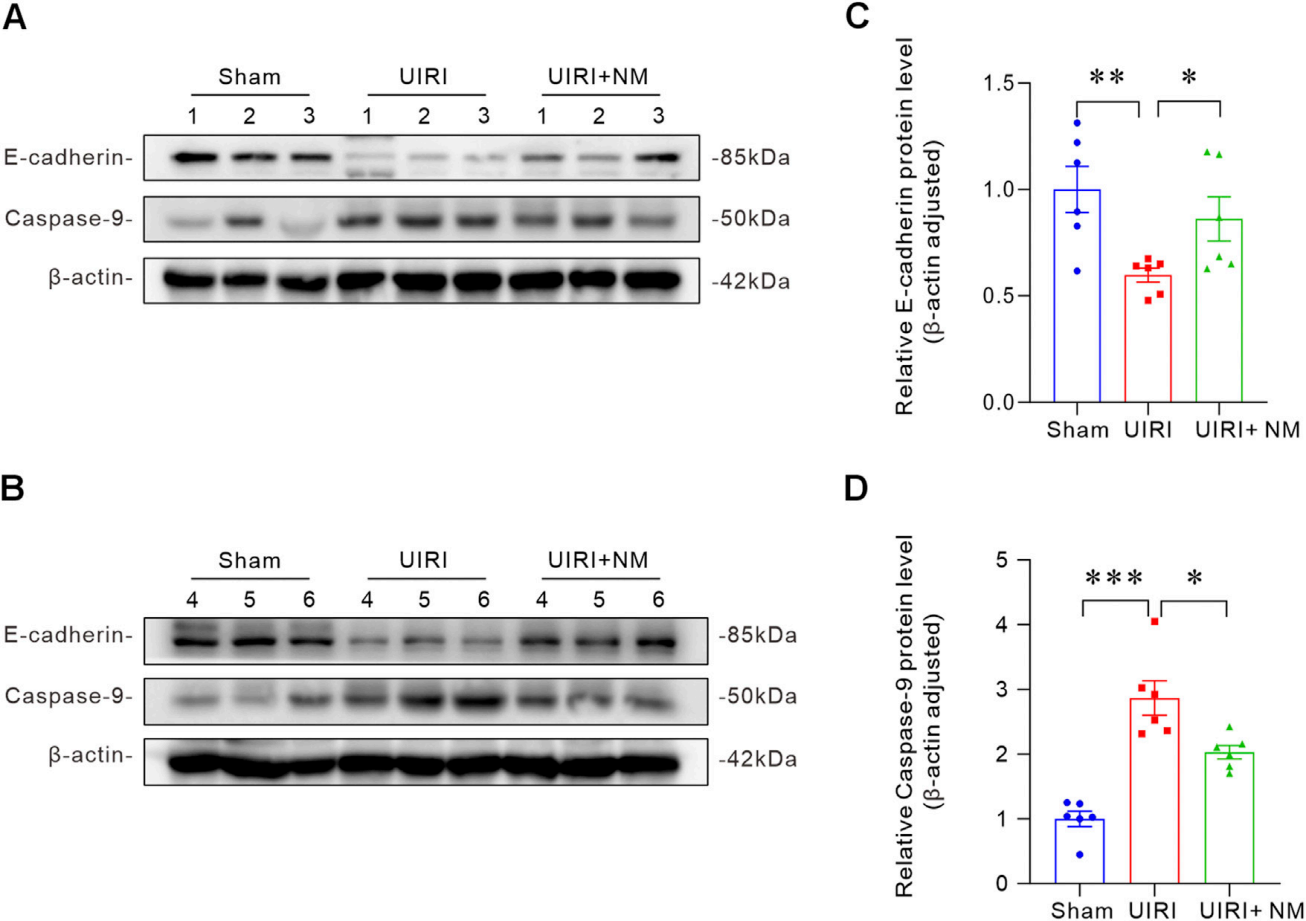
NM attenuated renal injury in UIRI mice. **(A,B)** Representative Western blots of E-cadherin and Caspase-9 demonstrating that NM treatment significantly alleviated renal injury in UIRI mice. **(C,D)** Quantitative analysis of E-cadherin **(C)** and Caspase-9 **(D)** expression (n = 6 biologically independent mice per group). *p < 0.05, **p < 0.01, ***p < 0.001. Data are presented as Mean ± SD. Statistical significance was determined by one-way ANOVA with Dunnett’s *post hoc* test.

To further confirm the protective effect of NM treatment on renal injury in UIRI mice, we analyzed the expression levels of E-cadherin, a surface marker of healthy tubular cells, and Caspase-9, a common marker of cellular injury and apoptosis, by Western blot (Figures 8A,B). Compared with the control group, the UIRI group showed significantly reduced E-cadherin protein levels, which were restored by NM treatment (Figure 8C), indicating that NM preserves renal epithelial integrity. Additionally, the UIRI group exhibited markedly increased Caspase-9 protein levels, while the NM-treated group showed a downward trend (Figure 8D), suggesting that NM inhibits apoptosis. The Western blot analysis revealed that the expression of TGF-β was significantly upregulates in the UIRI group, and the protein level recovered after NM treatment (Supplementary Figures S2A,B). These findings further confirm that the promotion of renal fibrosis is partially mediated through the activation of the TGF-β signaling pathway. In addition, we assessed the impact of ischemic injury on mitochondrial function and observed a marked downregulation of PGC-1α expression in the UIRI group. Notably, NM treatment restored the expression of PGC-1α, suggesting that NM can protect the kidneys by improving mitochondrial function (Supplementary Figures S2A–C). H&E staining showed that the structure of renal tubular was significantly damaged after UIRI, and NM treatment could improve this phenomenon (Supplementary Figures S2D,E). In summary, UIRI leads to significant apoptosis, loss of epithelial characteristics, pro-fibrotic process, and mitochondrial function impairment. NM treatment can effectively improve these pathological changes and demonstrate a good protective effect on the kidneys.

### NM inhibited the activation of IL-17/c-Fos signaling pathway

**FIGURE 9:**
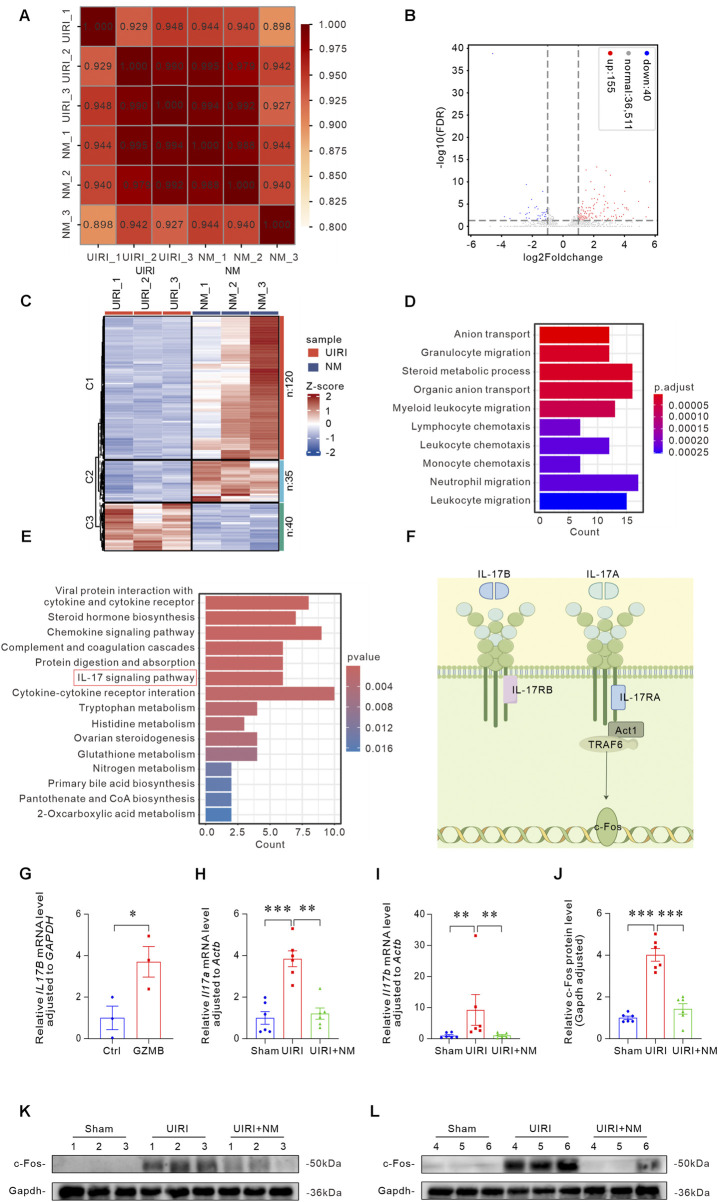
NM inhibited the activation of IL-17/c-Fos signaling pathway. **(A)** Correlation heatmap between UIRI and NM samples, with color intensity reflecting correlation strength. **(B)** Volcano plot of DEG distribution: blue dots represent downregulated genes, red dots indicate upregulated genes, and gray denotes non-differentially expressed genes. **(C)** Hierarchical clustering heatmap of DEGs across groups, with blue indicating low expression and red showing high expression genes. **(D)** Bar graph of GO functional enrichment analysis showing the top 10 most significant DEGs. **(E)** KEGG pathway enrichment analysis bar plot identifying the IL-17 signaling pathway as significantly altered. **(F)** Simplified schematic diagram of IL-17A/IL-17B signaling pathways. **(G)** qRT-PCR analysis of *IL17B* mRNA expression in HK-2 cells co-cultured with GZMB (5 ng/mL) (n = 3 biologically independent cell cultures). **(H,I)** Gene expression levels of *Il17a* **(H)** and *Il17b* **(I)** in kidneys of NM-treated and UIRI mice, demonstrating NM’s anti-inflammatory effects. **(J–L)** Quantitative analysis **(J)** and representative Western blots **(K,L)** of c-Fos expression (n = 6 biologically independent mice). *p < 0.05, **p < 0.01, ***p < 0.001. Data presented as Mean ± SD. Intergroup differences were assessed by one-way ANOVA followed by Dunnett’s *post hoc* test.

To elucidate the molecular mechanisms underlying NM’s therapeutic effects, we performed RNA-seq analysis on renal tissues. As shown in Figure 9A, high correlation coefficients between replicate samples confirmed experimental reproducibility. We identified 155 upregulated and 40 downregulated DEGs in NM-treated mice compared to UIRI controls (Figure 9B). Heat map revealed significant gene expression differences between UIRI and NM groups (Figure 9C). Functional enrichment analysis of DEGs identified the top 10 most significantly enriched GO terms, suggesting a prominent enrichment of genes associated with inflammatory cell chemotaxis (Figure 9D). KEGG pathway analysis of the 15 most prominent DEGs highlighted the IL-17 signaling pathway for further investigation (Figure 9E). The IL-17 signaling pathway activates the downstream Act1-TRAF6 complex via IL-17A binding to IL-17RA, leading to the induction of c-Fos (Figure 9F). In GZMB-stimulated HK-2 cells, *IL17B* mRNA expression was significantly elevated after 24 h (Figure 9G). Correspondingly, *in vivo* studies demonstrated altered expression of both *Il17a* (Figure 9H) and *Il17b* (Figure 9I). Western blot analysis confirmed that NM treatment reduced c-Fos protein expression in UIRI kidneys (Figures 9J–L). These results demonstrate that NM exerts renal protection by inhibiting the IL-17/c-Fos signaling pathway.

## Discussion

In this study, we observed an upregulation of GZMB expression in both *in vivo* renal fibrosis models and HK-2 cells treated with TGF-β. Treatment of human renal tubular epithelial cells with human recombinant protein GZMB not only induced cellular damage but also activated pro-inflammatory and pro-fibrotic pathways. Furthermore, GZMB induced tubular epithelial cell death in perforin-dependent and independent way. Importantly, we discovered the efficacy of NM in ameliorating renal injury and fibrosis, thereby providing a novel strategy for the treatment of CKD. This study highlights that NM exerts anti-fibrotic effects through targeted inhibition of the IL-17/c-Fos signaling pathway. IL-17A has been demonstrated to activate myofibroblasts and promote ECM, playing a pivotal role in multi-organ fibrosis. The research not only elucidates the biological significance of GZMB as a therapeutic target for CKD intervention, but also validates NM’s mechanistic actions, demonstrating its therapeutic potential for fibrotic diseases.

A key finding of this study is that we observed, for the first time, a significant increase in GZMB expression in injured HK-2 cells. Previous studies on GZMB in kidney diseases primarily emphasized its elevated expression in CD8^+^ T cells (Mueller et al., 2023; Jia et al., 2025). Notably, exogenous stimulation with GZMB in HK-2 cells further exacerbated cellular injury, upregulated fibrosis-related markers, and increased the release of inflammatory mediators, indicating its multifaceted role in renal damage.

As a member of the serine protease family, GZMB exhibits unique functions both intracellularly and extracellularly. Previous studies have primarily focused on its role as an immune effector molecule, inducing target cell apoptosis in a perforin-dependent manner (Froelich et al., 2009). Perforin, a pore-forming protein secreted by NK cells and CTLs, creates transmembrane pores in target cell membranes, facilitating the delivery of granzymes (House et al., 2017). In the classical cytotoxic function of GZMB, it is secreted alongside perforin by cytotoxic cells, enters target cells through perforin-formed pores, and initiates apoptosis (Thompson and Cao, 2024). In this study, we further discovered that GZMB, in synergy with perforin, not only induces apoptosis but also promotes ferroptosis. Additionally, GZMB possesses perforin-independent extracellular functions (Nüssing et al., 2022). According to published papers, intracellular GZMB can directly cleave executioner caspases such as caspase-3 and caspase-7, converting them from inactive zymogen forms into active proteases, thereby directly initiating the execution phase of apoptosis (Atkinson et al., 1998). Furthermore, we added recombinant GZMB protein to HK-2 cells and demonstrated that extracellular GZMB can directly cause damage to HK-2 cells. Further experiments showed that extracellular GZMB, with the assistance of perforin, significantly reduced cell numbers. Transcriptomic analysis suggests that the underlying mechanisms may involve apoptosis and ferroptosis. The molecular mechanism of apoptosis includes perforin forming pores on the target cell membrane, allowing GZMB to enter the cytoplasm and directly cleave and activate caspases (Berjis et al., 2024). However, the mechanism by which GZMB regulates ferroptosis has not been reported. It is reported that GZMB induces the production of reactive oxygen species (ROS). A possible mechanism by which GZMB promotes ferroptosis is through enhancing ROS generation, subsequently leading to increased lipid peroxidation (Jacquemin et al., 2015). Recent studies have revealed GZMB involvement in fibrosis across multiple organs, including the heart (Zeglinski and Granville, 2020; Santos-Zas et al., 2021), lungs (Kim et al., 2013), liver (Kellerer et al., 2024), adipose tissue (Cimini et al., 2020), and skin (Turner et al., 2019). Upregulated GZMB expression has been detected in fibrotic human and murine hearts, where it positively correlates with inflammation, myofibroblast activation, and fibrosis severity in a perforin-independent manner (Shen et al., 2016). In chronic obstructive pulmonary disease (COPD), GZMB is highly expressed in type II alveolar epithelial cells (Ngan et al., 2009), monocytes, and granulocytes, indicating its role as a key mediator in non-immune cells. Similarly, elevated GZMB levels and increased GZMB-expressing CD8^+^ T cells have been observed in the bronchoalveolar lavage (BAL) of COPD patients. CD8^+^ T cell-derived granzymes can influence ECM degradation and remodeling, suggesting that GZMB upregulation contributes to airway remodeling by promoting ECM deposition (Kim et al., 2013). Furthermore, GZMB plays a crucial role in skin and adipose tissue fibrosis through multiple pathways, including the release of active TGF-β regulators, enhancement of pro-inflammatory cytokine activity, epithelial-mesenchymal transition (EMT), and ECM remodeling (Turner et al., 2019; Turner et al., 2021; Grøndal et al., 2024). However, research on the impact of GZMB on renal injury and fibrosis remains limited. In this study, GZMB overexpression in HK-2 cells induced the expression of α-SMA, collagen I, and fibronectin, along with pro-inflammatory effects (upregulation of IL-1β, IL-6, IL-8, and TNF-α). These findings highlight the dual pro-fibrotic and pro-inflammatory roles of GZMB in kidney diseases. It is well known that podocytes and mesangial cells are two specialized cell types in the glomerulus that play critical roles in kidney diseases (Kuo et al., 2022), yet their relationship with GZMB remains unclear. While this study focuses on the effects of GZMB on tubular cells, investigating its role in podocytes and mesangial cells represents a promising direction for future research.

Another intriguing aspect of this study is the first demonstration of NM’s anti-fibrotic effects in the kidney. Previous studies on NM in the kidney have primarily focused on its anticoagulant properties during continuous renal replacement therapy, while its renal fibrosis remains largely unexplored. Here, our study successfully demonstrates that NM exhibits inhibitory effects in both *in vivo* and *in vitro* renal fibrosis models. We found that the concentration of NM at 100 μM had no significant effect on HK-2 cell activity. The results indicate that the protective effects in co-treatment are not confounded by intrinsic toxicity of NM. And, we found that NM effectively reversed the upregulation of TGF-β expression and the downregulation of PGC-1α expression induced by UIRI, indicating that it can inhibit the fibrosis signaling pathway activator and improve mitochondrial function. Meanwhile, the histopathological results demonstrated that NM treatment effectively attenuated renal tubular structural damage. These fundings provide conclusive evidence supporting the therapeutic efficacy of NM. Furthermore, we discovered that NM can suppress the activation of the IL-17/c-Fos signaling pathway, indicating that its renal protective effects are mediated not only through anti-fibrotic mechanisms but also via anti-inflammatory functions. As a multitarget serine protease inhibitor, plasminogen activator inhibitor-1(PAI-1) plays a pro-fibrotic function in both UUO and diabetic nephropathy models, primarily mediated by inhibiting tissue-type plasminogen activator (tPA) and urokinase-type plasminogen activator (uPA) thereby promoting ECM deposition and suppressing fibrinlysis (Gu et al., 2016; Yao et al., 2019). In contrast, kallistatin ameliorates renal fibrosis by attenuating EMT and fibroblast activation through inhibition of the Wnt/β-catenin and TGF-β signaling pathway (Yiu et al., 2021; Yuan et al., 2022b). Therefore, the protective effect of NM may be mediated through serine proteases or modulating related signaling pathway, such as suppressing thrombin activity to reduce blood-brain barrier disruption after spinal cord injury and inhibiting the NF-κB signaling pathway to exert anti-inflammatory effects (Jha et al., 2021; Zhao et al., 2022; Chen et al., 2023). Based on existing literature, perforin expression is known to be upregulated by the transcription factor NF-κB (Zhou et al., 2002). NM significantly inhibits the activation of NF-κB (Sendler et al., 2018). Therefore, it is plausible that NM may indirectly downregulate perforin expression via suppression of the NF-κB pathway. Additionally, NM blocks kallikrein to diminish downstream effects, such as inhibiting brdykinin-mediated inflammation—a therapeutic pathway for sepsis and a potential mechanism for mitigating inflammation during cardiopulmonary bypass (CPB) (He et al., 2024). As a serine protease inhibitor, NM’s anti-fibrotic function is not solely mediated by GZMB inhibition but may also involve the suppression of other serine proteases.

Furthermore, this study reveals that the IL-17/c-Fos signaling pathway plays a pivotal role in NM’s therapeutic effects on ischemic kidney disease. Lin et al. (2014) demonstrated that NM treatment significantly reduced IL-17A expression levels in both bronchoalveolar lavage fluid and lung tissues of mice with chronic asthma. In a rhabdomyolysis-induced acute kidney injury model, KEGG pathway analysis showed that NM treatment downregulated the IL-17 signaling pathway (Guo et al., 2022). The above results are consistent with the findings of the present study. Recent studies have indicated that IL-17 drives fibrosis in multiple organs (e.g., heart (Zhang et al., 2018), liver (Beringer and Miossec, 2018), and lungs (Celada et al., 2018)). In kidney diseases, Peng et al. (2015) observed upregulated IL-17A expression in an obstructive nephropathy model, while IL-17A knockout mice exhibited significantly attenuated myofibroblast activation, ECM deposition, and inflammatory responses. These findings further underscore the critical role of IL-17 in renal inflammation and fibrosis.

In conclusion, our study demonstrates that GZMB plays a crucial role in renal injury and interstitial fibrosis. Exogenous GZMB stimulation of HK-2 cells promotes cellular damage, inflammatory responses and p-EMT, suggesting GZMB as a promising therapeutic target for intervention. Moreover, our investigation of the serine protease inhibitor NM provides the first evidence of its anti-fibrotic potential, identifying NM as a novel therapeutic candidate for CKD.

## Data Availability

The data presented in the study are deposited in the GSA repository, accession numbers CRA031364, HRA013864.
