# Correction: Nafamostat mesilate attenuates renal fibrosis by suppressing the IL-17 signaling pathway

**DOI:** 10.3389/fphar.2025.1764700

**Published:** 2026-01-02

**Authors:** Weili Liao, Rui Fan, Yuman Du, Hairong Wang, Yong Yang, Yuan Tian

**Affiliations:** 1 Department of Nephrology, Jingzhou Hospital Affiliated to Yangtze University, Jingzhou, China; 2 School of Public Health, Mudanjiang Medical University, Mudanjiang, China

**Keywords:** renal interstitial fibrosis, Granzyme B, nafamostat mesylate, tubular injury, inflammation

The order of **Affiliations** was incorrect. The correct order is:

1 Department of Nephrology, Jingzhou Hospital Affiliated to Yangtze University, Jingzhou, China.

2 School of Public Health, Mudanjiang Medical University, Mudanjiang, China.

The original article has been updated.

