# Supplementary material for: Nafamostat mesilate attenuates renal fibrosis by suppressing the IL-17 signaling pathway
Source: Front Pharmacol. 2025 Oct 31;16:1648623. doi: 10.3389/fphar.2025.1648623 (PMC12615172; doi:10.3389/fphar.2025.1648623)
Supplement: Supplementary file 1 [file Table1.docx]

**Table S1 List of antibodies**

| Primary antibodies | Host | Dilution(Application)and supplier |
| --- | --- | --- |
| GzmB | Rabbit | 1:1000(WB) 13588-1-AP,Proteintech |
| Ki-67 | Rabbit | 1:1000(WB) 27309-1-AP,Proteintech |
| Ngal | Rabbit | 1:1000(WB) 26991-1-AP,Proteintech |
| Fibronectin | Rabbit | 1:8000(WB)1:100(IF) F3648,Merck Sigma-Aldrich |
| α-SMA | Rabbit | 1:1000(WB)1:100(IF)  14395-1-AP,Proteintech |
| Collagen I | Rabbit | 1:1000(WB) ab138492,abcam |
| E-cadherin | Mouse | 1:4000(WB) 60335-1-Ig,Proteintech |
| Caspase-9 | Rabbit | 1:1000(WB) 10380-1-AP,Proteintech |
| c-Fos | Mouse | 1:5000(WB) 66590-1,Proteintech |
| α-tubulin | Mouse | 1:10000(WB) 66031-1-Ig,Proteintech |
| β-actin | Rabbit | 1:10000(WB) 20536-1-AP,Proteintech |
| Gapdh | Rabbit | 1:10000(WB) 10494-1-AP,Proteintech |

**Table S2 Primers for qRT-PCR**

| Gene | Species | Forward(5’to3’) | Reverse(5’to3’) |
| --- | --- | --- | --- |
| *LCN2* | Human | CTGAGTGCACAGGTGCCG | TTTAGCAGACAAGGTGGGGC |
| *HAVCR1* | Human | TCAGCTCGGGAATGCACA | TGGTTGCCTTCCGTGTCT |
| *IL1B* | Human | TGCCACCTTTTGACAGTGATG | AAGGTCCACGGGAAAGACAC |
| *Il6* | Human | GTGGCTAAGGACCAAGACCA | GGTTTGCCGAGTAGACCTCA |
| *Il8* | Human | AGAGTGGACCACACTGCGC | ACATCCCAACGGTCTACGTTA |
| *TNF* | Human | ACGTCGTAGCAAACCACCAA | GCAGCCTTGTCCCTTGAAGA |
| *TGFB1* | Human | GCAACAATTCCTGGCGTTAC | GCTGAATCGAAAGCCCTGTA |
| *FN1* | Human | CCGCCGAATGTAGGACAAGA | TGCCTCTGCTGGTCTTTCAG |
| *ACTA2* | Human | GCTACCCGCCCAGAAACTA | CCAGTTGGTGATGATGCCGT |
| *COL1A1* | Human | GTGCGATGACGTGATCTGTGA | CGGTGGTTTCTTGGTCGGT |
| *IL17B* | Human | CTCCTGCTTCTAGGCTGGTTG | CCACCTGGCACTTCGAGTTAG |
| *GAPDH* | Human | GACAGTCAGCCGCATCTTCT | GCGCCCAATACGACCAAATC |
| *ACTB* | Human | AGCTGCGTTTTACACCCTTT | AAGCCATGCCAATGTTGTCT |
| *Fn1* | Mouse | GATGAGCTTCCCCAACTGGT | CTGGGTTGTTGGTGGGATGT |
| *GzmB* | Mouse | GCTCTGATTACCCATCGTCCC | GGCAGAAGAGGTGTTCCATTG |
| *Col1a1* | Mouse | ATCTCCTGGTGCTGATGGAC | ACCTTGTTTGCCAGGTTCAC |
| *Prf* | Mouse | TGGGTCTACAGGGTCTCAAA | ATGTTTACGCTTCGTGGCAG |
| *Il17a* | Mouse | TCCCTCTGTGATCTGGGAAG | CTCGACCCTGAAAGTGAAGG |
| *Il17b* | Mouse | CTTGGTGGGGGGGGACTGG | TCTTTTGCCTTTGGTGTTCC |
| *Gapdh* | Mouse | AAATGGTGAAGGTCGGTGTGAAC | CAACAATCTCCACTTTGCCACTG |
| *Actb* | Mouse | CAGAAGGAGATTACTGCTCTGGCT | TACTCCTGCTTGCTGATCCACATC |
