# Supplementary material for: Nafamostat mesilate attenuates renal fibrosis by suppressing the IL-17 signaling pathway
Source: Front Pharmacol. 2025 Oct 31;16:1648623. doi: 10.3389/fphar.2025.1648623 (PMC12615172; doi:10.3389/fphar.2025.1648623)
Supplement: Supplementary file 2 [file DataSheet1.docx]

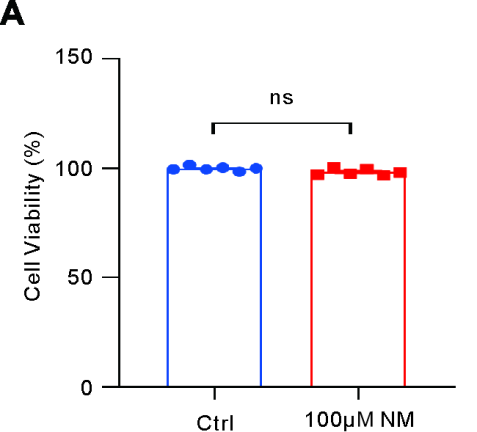


**Figure S1 NM with a concentration of 100μM had no significant effect on HK-2 cell activity.** (A) CCK8 evaluated the activity of HK-2 cells stimulated by 100μM concentration of NM (n=3 per group). ns p>0.05. Data are presented as Mean ± SD, conforming to normal distribution and homogeneity of variance. Intergroup comparisons were performed using Two-independent sample t-test.


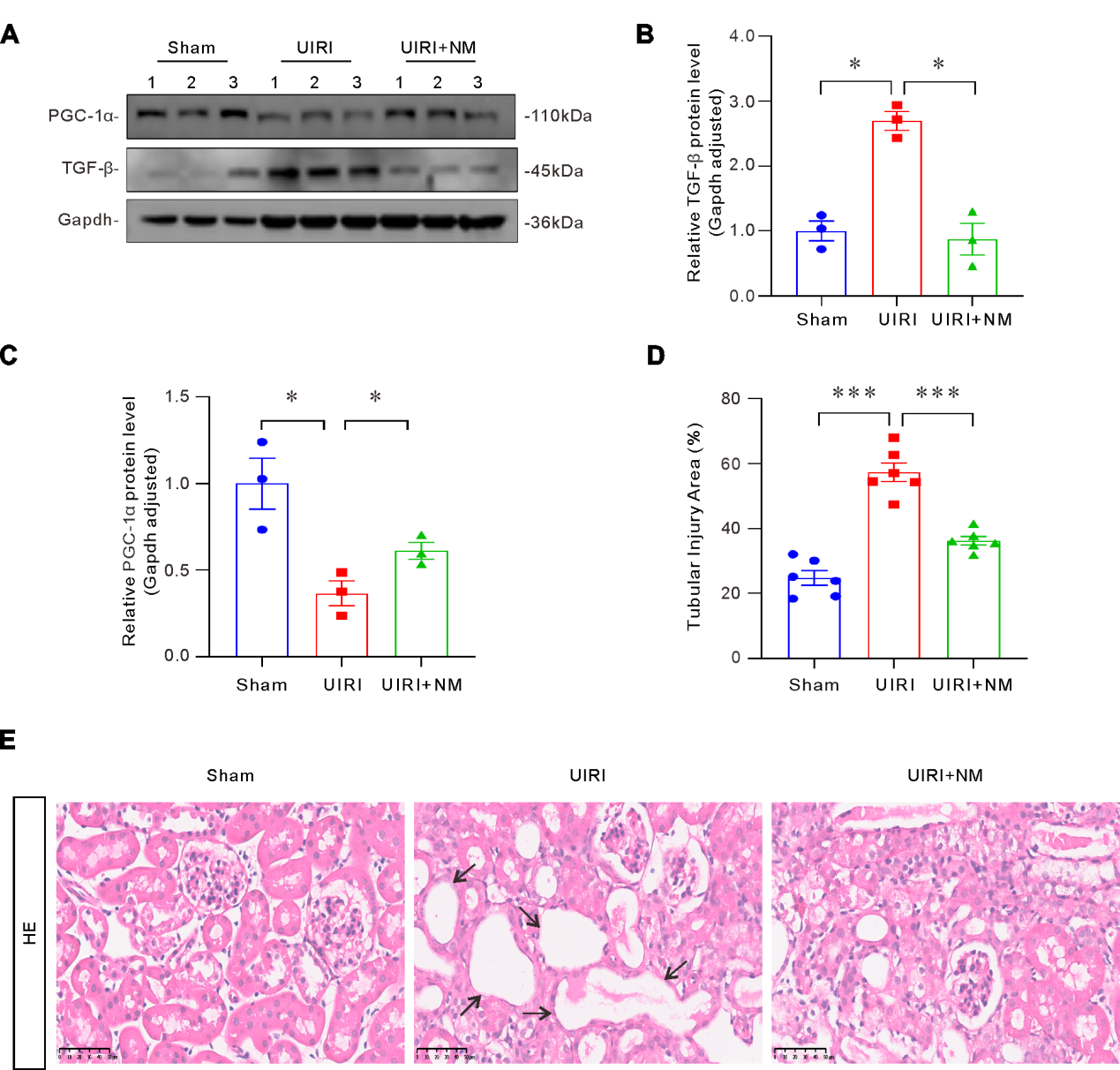


**Figure S2 NM attenuated renal injury in UIRI mice.** (A-C) Representative Western blot (A) and quantitative analysis (B-C) of PGC-1α and TGF-β protein levels in the kidneys of different groups (n=3 biologically independent mice). (D-E) Representative H&E staining section of mouse kidney tissue (scale bar: 50 μm) and quantitative analysis of the injurious area percentage (n=6 biologically independent mice) and arrow marks pointed to tubular atrophy and dilated tubules. *p<0.05, ***p<0.001. Data are presented as Mean ± SD. Intergroup differences were analyzed by one-way ANOVA followed by Bonferroni or Dunnett post-hoc tests.
